# Efficacy of clonidine as an adjuvant to bupivacaine for caudal analgesia in children undergoing sub-umbilical surgery

**DOI:** 10.4103/0019-5049.71047

**Published:** 2010

**Authors:** Aruna Parameswari, Anand M Dhev, Mahesh Vakamudi

**Affiliations:** Department of Anaesthesiology, Critical Care and Pain Medicine, Sri Ramachandra University, Porur, Chennai, India

**Keywords:** Bupivacaine, caudal analgesia, clonidine, post-operative analgesia, sub-umbilical surgery

## Abstract

Caudal epidural analgesia with bupivacaine is very popular in paediatric anaesthesia for providing intra- and postoperative analgesia. Several adjuvants have been used to prolong the action of bupivacaine. We evaluated the efficacy of clonidine added to bupivacaine in prolonging the analgesia produced by caudal bupivacaine in children undergoing sub-umbilical surgery. One hundred children, age one to three years, undergoing sub-umbilical surgery, were prospectively randomized to one of two groups: caudal analgesia with 1 ml/kg of 0.25% bupivacaine in normal saline (Group A) or caudal analgesia with 1 ml/kg of 0.25% bupivacaine with 1 µg/kg of clonidine in normal saline (Group B). Post-operative pain was assessed for 24 hours using the FLACC scale. The mean duration of analgesia was significantly longer in Group B (593.4 ± 423.3 min) than in Group A (288.7 ± 259.1 min); *P* < 0.05. The pain score assessed using FLACC scale was compared between the two groups, and children in Group B had lower pain scores, which was statistically significant. The requirement of rescue medicine was lesser in Group B. Clonidine in a dose of 1 µg/kg added to 0.25% bupivacaine for caudal analgesia, during sub-umbilical surgeries, prolongs the duration of analgesia of bupivacaine, without any side effects.

## INTRODUCTION

Caudal analgesia is an accepted and popular method of providing intra- and post-operative analgesia for abdominal, perineal and lower limb surgeries in children. Bupivacaine is the most commonly used local anaesthetic for this purpose. The limitation of bupivacaine is the short duration of action, about four to six hours, when administered as a ‘single shot technique’. Several adjuncts such as opioids, ketamine, midazolam, clonidine and neostigmine have been used with bupivacaine to prolong its action,[1–5] and thus extend the duration of post-operative analgesia provided by the ‘single shot’ caudal technique. Clonidine, an alpha 2 agonist has extensively been used in neuraxial blocks[6–9] and peripheral nerve blocks to prolong the action of bupivacaine. It is one of the most commonly used additives with bupivacaine for caudal analgesia in children.[10] However, the role of clonidine in improving and prolonging the analgesia produced by caudal bupivacaine is highly variable in different published studies. Also, the duration of post-operative analgesia using caudal clonidine bupivacaine mixtures is also highly variable. We conducted this study to assess the efficacy of clonidine in prolonging the action of bupivacaine when used for caudal epidural analgesia in children undergoing sub-umbilical surgeries.

## METHODS

After obtaining institutional ethical committee approval and informed consent, this prospective, randomised, controlled, double-blinded, single-centre study was conducted in 100 patients, ASA physical status I – II, age one to three years, undergoing sub-umbilical surgeries under general anaesthesia. Children with sacral bone abnormalities, spina bifida, coagulopathy and infection at the site of caudal injection were excluded from the study.

The sample size was determined by power analysis. The patients were randomly allocated into two groups: Group A (Control group) and Group B (Study group). Randomisation was done by picking random lots from a sealed bag. Twenty minutes before shifting them to the operating room, oral midazolam 0.5 mg/kg was administered as pre-medication to all the children in both the groups. The patients were then shifted into the operating room and connected to monitors; electrocardiogram, non-invasive blood pressure and pulse oximeter, and baseline values were recorded. The children were induced with 50% nitrous oxide, 50% oxygen and 8% sevoflurane. Intravenous access was secured and lactated Ringer’s solution was administered as per the calculated fluid requirements. Inj. Fentanyl 2 µg/kg was given intravenously for analgesia. Airway management was left to the discretion of the attending anaesthesiologist and the children were managed with face mask, laryngeal mask airway or endotracheal tube, with or without muscle relaxants. After induction, patients were placed in the lateral decubitus position and a single shot caudal block was performed, with aseptic precautions, using a 23 G hypodermic needle, by an anaesthesiologist who was blinded to the drug that was to be administered in the caudal epidural space. The drug was loaded by an anaesthesiologist who did not participate in the study. Group A patients received 1 ml/kg of 0.25% bupivacaine in normal saline and Group B patients received 1 ml/kg of 0.25% bupivacaine with 1 µg/kg of clonidine in normal saline. The patients were extubated at the end of the procedure and the duration of anaesthesia was noted in both the groups. The patients were assessed for 24 hours post-operatively. Post-operative assessment was done by another anaesthesiologist in the post-anaesthesia care unit (PACU) who was not aware of the drug administered and by a nurse in the ward who was also blinded. Physiological measures assessed were heart rate and blood pressure for two hours in the post-anaesthesia care unit. Pain score was assessed using the FLACC (F – Face, L – Leg, A – Activity, C – Cry, C – Consolability) scale [Table 1]. The severity of the pain was classified using the total FLACC score as given herewith:

**Table 1:** The FLACC scale for pain assessment in children. There are five parameters, each given a score of 0–2 and the total score is taken to assess pain

Category	Scoring		
	0	1	2
Face	No particular expression or smile	Occasional grimace or frown, withdrawn, disinterested	Frequent to constant quivering chin, clenched jaw
Legs	Normal position or relaxed	Uneasy, restless, tense	Kicking or legs drawn up
Activity	Lying quietly, normal position, moves easily	Squirming, shifting back and forth, tense	Arched, rigid or jerking
Cry	No cry (awake or asleep)	Moans or whimpers; occasional complaint	Crying steadily, screams or sobs, frequent complaints
Consolability	Content, relaxed	Reassured by occasional touching, hugging or being talked to; distractable	Difficult to console

Each of the five categories is scored from 0 – 2, resulting in total range of 0 – 10, FLACC = Face, Leg, Activity, Cry, Consolability

0 = No pain
1 - 3 = Mild pain
4 - 7 = Moderate pain
8 - 10 = Severe pain

The heart rate and blood pressure were measured at 15, 30, 45, 60, 90 and 120 minutes post-operatively. Assessment of pain by FLACC scale was done at 0, 1, 2, 6, 12 and 24 hours post-operatively.

The time from arrival in the post anaesthesia care unit to the first time the FLACC score was greater than 4 was recorded and noted as the duration of adequate caudal analgesia.

In the post anaesthesia care unit, the necessity for rescue medicine was decided by the pain score. Rescue medication was administered when the FLACC score was ≥ 4. Paracetamol suppository was used as rescue medicine with a loading dose of 40 mg/kg followed by 20 mg/kg every six hours.

The number of doses of rescue medication required and the time to first administration of rescue medication were also noted.

In the post-operative period, patients were also monitored for adverse effects, including respiratory depression, vomiting, hypotension and bradycardia. Respiratory depression was defined as a decrease in oxygen saturation less than 93%, requiring oxygen by face mask. Hypotension was defined as systolic blood pressure less than 70 mm Hg and bradycardia was defined as a heart rate less than 80 beats/min.

## RESULTS

Analysis of patient results revealed no differences in the demographic characteristics of the two groups [Table 2].

**Table 2:** General characteristics of the patients and the duration of surgery in the two groups

Variable	Group A (Plain bupivacaine)	Group B (Bupivacaine with 1 µg/kg clonidine)
Number of patients	50	50
Age (months)	21	19
Male (number)	47	47
Female (number)	3	3
Weight (kg)	10	9
Duration of surgery (minutes)	29	26

The mean age of the children in the two groups were compared using independent T-test. Group A patients had a mean age of 20.72 months and Group B patients had a mean age of 19.32 months. The difference was insignificant with a *P* value of 0.253. There was no significant difference between the two groups in terms of weight, sex, type of surgery and duration of surgery. The type of surgery included circumcision, herniotomy and orchidopexy and it was equally distributed between the two groups [Table 3].

**Table 3:** Type of surgery in Group A (Plain bupivacaine) and Group B (Clonidine and bupivacaine)

Type of surgery	Group A	Group B
Circumcision	17	15
Orchidopexy	7	6
Herniotomy	26	29

There was no significant difference in the intra-operative and post-operative heart rate and blood pressure between the two groups. The children in Group A had a mean post-operative heart rate of 129.96 ± 14.47 beats/min, while children in Group B had a mean post-operative heart rate of 123.62 ± 15.14 beats/min. The difference was not statistically significant.

The children in Group A had a postoperative systolic blood pressure of 105.92 ± 6.23 mm Hg (mean ± SD), while the children in Group B had a post-operative systolic blood pressure of 105.38 ± 9.11 mm Hg, and the difference was not statistically significant.

The pain score was assessed using the FLACC scale and the two groups were compared using Pearsons Chi-square test. It was found that there was a significant difference between the two groups from two hours to six hours post-operatively with a *P* value < 0.05 [Table 4]. Two hours post-operatively, 13 patients (26%) in Group A had moderate-to-severe-pain (pain score 4 – 10), while only three patients (6%) in Group B had moderate-to-severe pain (pain score 4 – 10) and the difference was statistically significant; *P* value of 0.009. At six hours post-operatively, 38 patients (76%) in Group A and 17 patients (34%) in Group B had moderate-to-severe pain (4-10 pain score) and the difference was statistically significant; *P* value of 0.000.

**Table 4:** Pain scores calculated using the FLACC scale in both the groups. More children in Group A (Plain bupivacaine) had moderate-to-severe pain at 2 hours and 6 hours post-operatively, compared to children in Group B (Clonidine with bupivacaine)

Pain score at 2 hours	0 – 3 (No pain or mild pain) n (%)	4 – 10 (moderate-tosevere pain) n (%)
Group A	37 (74)	13 (26)
Group B	47 (94)	3 (6)
**Pain score at 6 hours**		
Group A	12 (24)	38 (76)
Group B	33 (66)	17 (34)

**Figure 1:**
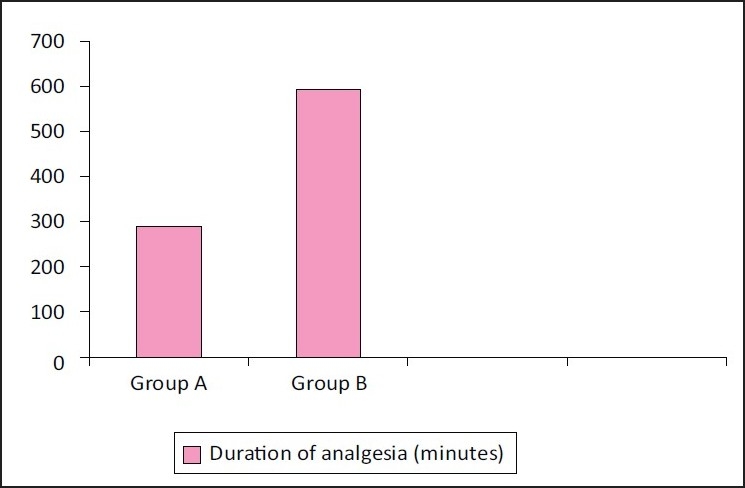
Duration of analgesia in both the groups. Group A received 1 ml/kg of 0.25% plain bupivacaine and Group B received 1 ml/kg of 0.25% bupivacaine containing 1 µg/kg clonidine. The mean duration of analgesia was 288.7 minutes in Group A and 593.4 minutes in Group B

The duration of analgesia between the two groups were compared using Mann-Whitney test. Group A had a mean duration of analgesia of 288.7 ± 259.1 min and Group B had a mean duration of analgesia of 593.4 ± 423.3 min and the difference was statistically significant with a *P* value of 0.000 [Figure 1].

**Figure 2:**
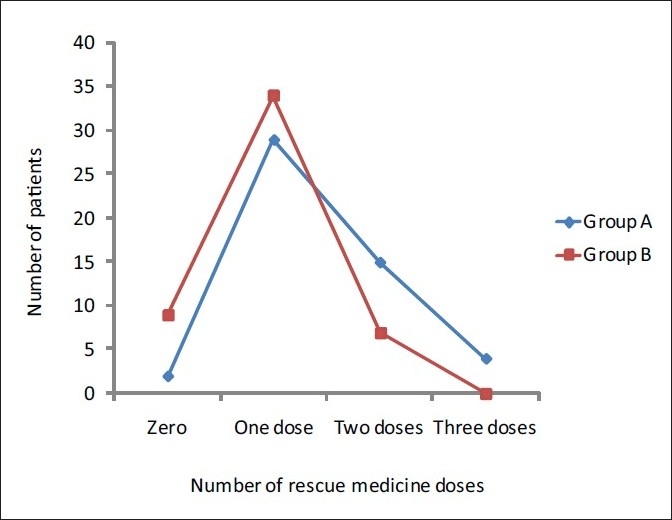
Number of doses of rescue medicine in the two groups. More patients in Group A (Plain bupivacaine) needed two or three doses of rescue medicines compared to patients in Group B (Clonidine with bupivacaine)

The requirement of rescue medicine was compared between the two groups using Pearsons Chi-square test and it was found to be significant with a *P* value of 0.008, with nine children not requiring rescue analgesia for 24 hours post-operatively in Group B compared to two children not requiring rescue analgesia for 24 hours post-operatively in Group A [Figure 2].

None of the children in the two groups had respiratory depression, hypotension, bradycardia or vomiting.

## DISCUSSION

Our study indicates that addition of clonidine 1 µg/kg to bupivacaine 0.25% for caudal analgesia in children, significantly prolongs the duration of analgesia, as compared with Bupivacaine alone. Our findings are consistent with those reported by several other studies.[6–9,11–13]

Several adjuvants have been used to prolong the duration of analgesia of bupivacaine for caudal analgesia in children. Opioids, ketamine and midazolam are some of the commonly used drugs[11,12,14] The use of opioids is associated with an increased incidence of pruritus and post-operative nausea and vomiting.[14] The advantage of clonidine is that it prolongs the duration of analgesia without an increase in the incidence of respiratory depression, pruritus and urinary retention which are commonly seen with neuraxial opioids.

We used oral midazolam pre-medication in our children as it is one of the most commonly used pre-medicants and it is our hospital policy to administer it as a pre-medicant in children older than six months. We also wanted to keep the pre-medication uniform in all children to avoid the confounding effect of the pre-medicant drug in assessment of post-operative analgesia.

We chose the FLACC scale to evaluate pain post-operatively as it is easy to use, is validated and gives us an objective evaluation.[15]

We chose to monitor our patients for a period of 24 hours post-operatively. This is in contrast to a few other studies where there was only a six-hour period of observation post-operatively[16–17] and the rest of the assessment was done by parents. Assessment by parents could introduce some inconsistency as parents differ in the way they perceive their children to be in pain and the threshold for administering rescue medications varies between parents.

In children, a mixture of 0.25% bupivacaine with 1 - 2 µg/kg clonidine has been seen to improve the duration and quality of analgesia provided by caudal block. Although results differ widely, the duration of analgesia provided range from 6.3 hours[12] to 16.4 hours[6] for 1 µg/kg to 5.8[11] and 9.8 hours[7] for 2 µg/kg^.^ One study has shown a mean duration of analgesia of 20.9 ± 7.4 hours in children receiving caudal clonidine with bupivacaine, but a dose of 5 µg/kg of clonidine was used in this study.[13] The wide variation in the duration of action of clonidine in the various studies could be due to many reasons: dose of clonidine used, differences in pre-medication and volatile anaesthetic used, type of surgery, indications for rescue analgesia, assessment of pain, and statistical analysis. The duration of analgesia in the clonidine group in our study was 10 hours, while that in the plain bupivacaine group was 4.5 hours, which was similar to other studies.

The mean duration of analgesia of clonidine is also increased when it is combined in a dose of 1 – 2 µg/kg with caudal S+ ketamine 1 mg/kg (22.7 hours and 21.8 hours, respectively, with 1 and 2 µg/kg).[18]

Several mechanisms have been suggested for the clonidine-induced prolongation of caudal analgesia with bupivacaine. The anti-nociceptive action is due to the direct suppression of the spinal cord nociceptive neurons by epidural clonidine. Another mechanism is that clonidine crosses the blood brain barrier and interacts with alpha 2 adrenoceptors at spinal and supraspinal sites to produce analgesia. Clonidine also suppresses neurotransmission in peripheral sensory A δ and C nerve fibres. The final mechanism suggested is pharmacokinetically mediated: clonidine induces vasoconstriction through α -2b adrenoceptors located at the peripheral vascular smooth muscles.[19]

The dose of clonidine for epidural administration is 1 – 5 µg/kg. We chose a dose of 1 µg/kg of clonidine in our study as there were studies showing that increasing the dose from 1 to 2 µg/kg did not enhance the analgesic efficacy of clonidine[17] and the incidence of adverse effects like respiratory depression, bradycardia and hypotension increased with increasing dose.[20]

Although many studies have supported the analgesic benefits of caudal clonidine as an additive, there are some studies that have shown that there is no such benefit.[21–24] Some studies have also shown that the incidence of vomiting is higher with caudal clonidine,[21] and some have shown that the mean time of arousal from anaesthesia is significantly prolonged.[23] None of the children in our study, who had received clonidine, had post-operative vomiting. One limitation of our study was that we did not assess the mean time of arousal from anaesthesia in both the groups.

Sharpe *et al*.[23] speculated that a small volume of bupivacaine (0.5 mL/kg) may not be enough to deliver clonidine up to the spinal cord, leaving only direct action on the nerve routes in the caudal area. These findings suggest that the addition of clonidine 2 µg/kg to low volumes of caudal anaesthetics has limited clinical benefit in children undergoing circumcision. This was the reason we had chosen a standard dose of 1 mL/kg of 0.25% bupivacaine as the final volume in all the children in both the groups.

The side effects of neuraxial clonidine administration include hypotension and bradycardia. The antihypertensive effect results from stimulation of α2 inhibitory neurones in the medullary vasomotor centre of the brainstem, which leads to a reduction in nor-epinephrine turnover and sympathetic nerve outflow from the CNS to the peripheral tissues. Bradycardia is caused by an increase in vagal tone resulting from central stimulation of parasympathetic outflow, as well as reduced sympathetic drive.[1]

In children, clonidine 1-5 µg/kg has been used without clinically significant respiratory or haemodynamic effects. Although haemodynamic side-effects appear to be less pronounced in children than in adults, they may be dose-dependent, as reported by Motsch and colleagues.[13] One case of life-threatening apnoea following inguinal herniorrhaphy and orchidopexy, in a two week-old term neonate, has been reported.[20] Many studies have reported a high incidence of these side effects, but none of the children in our study, who received clonidine, developed hypotension or bradycardia.

We conclude that clonidine in a dose of 1 µg/kg, added to 0.25% bupivacaine for caudal analgesia and administered as a 1ml/kg mixture in children, for sub-umbilical surgery, significantly prolongs the duration of post-operative analgesia when compared to 1ml/kg of 0.25% bupivacaine alone, without any side effects.
